# Beware of a Fragile Footplate: Lessons from Ossiculoplasty in Patients with Ossicular Anomalies Related to Second Pharyngeal Arch Defects

**DOI:** 10.3390/jcm8122130

**Published:** 2019-12-03

**Authors:** Sun A Han, Goun Choe, Yoonjoong Kim, Ja-Won Koo, Byung Yoon Choi, Jae-Jin Song

**Affiliations:** Department of Otorhinolaryngology-Head and Neck Surgery, Seoul National University Bundang Hospital, 300 Gumi-dong, Bundang-gu, Seongnam 13620, Korea; hsa226@gmail.com (S.A.H.); delicatewave@gmail.com (G.C.); mdyjkim@gmail.com (Y.K.); jwkoo99@snu.ac.kr (J.-W.K.); choiby@snubh.org (B.Y.C.)

**Keywords:** ossicular anomaly, footplate, ossiculoplasty, second pharyngeal arch

## Abstract

Background and objectives: We review the intraoperative findings and postoperative outcomes of ossiculoplasty in subjects with second pharyngeal arch (SPA)-derived ossicular anomalies. We summarize potential intraoperative complications and recommend precautions that may reduce the risk of fracture. Materials and Methods: Twenty-four patients with SPA-derived ossicular anomalies were included, and pre- and postoperative audiometric results were compared. Results: The mean air conduction threshold (56.0 ± 12.4 dB HL) was significantly improved 1 month (27.6 ± 10.1 dB HL) and 6 months (23.8 ± 13.2 dB HL) after surgery (*p* < 0.001). The preoperative air–bone gap (ABG) (40.4 ± 7.4 dB HL) was significantly decreased at 1 month (15.1 ± 5.9 dB HL) and 6 months (11.3 ± 8.9 dB HL) postoperation. ABG closure was successful (<20 dB HL) in 21 (87.5%) patients 6 months after surgery. Intraoperative footplate fractures occurred in 3 of 24 patients. The fractures were managed successfully, and the ABG closure was successful in all cases. Conclusions: The stapes footplate is likely to be relatively thin in subjects with SPA-derived ossicular anomalies because the footplate is partially or totally derived from the SPA. Thus, a fragile footplate should be expected, and care is needed when handling the footplate. However, when complications are overcome, the audiological outcomes are excellent in most cases.

## 1. Introduction

**Figure 1:**
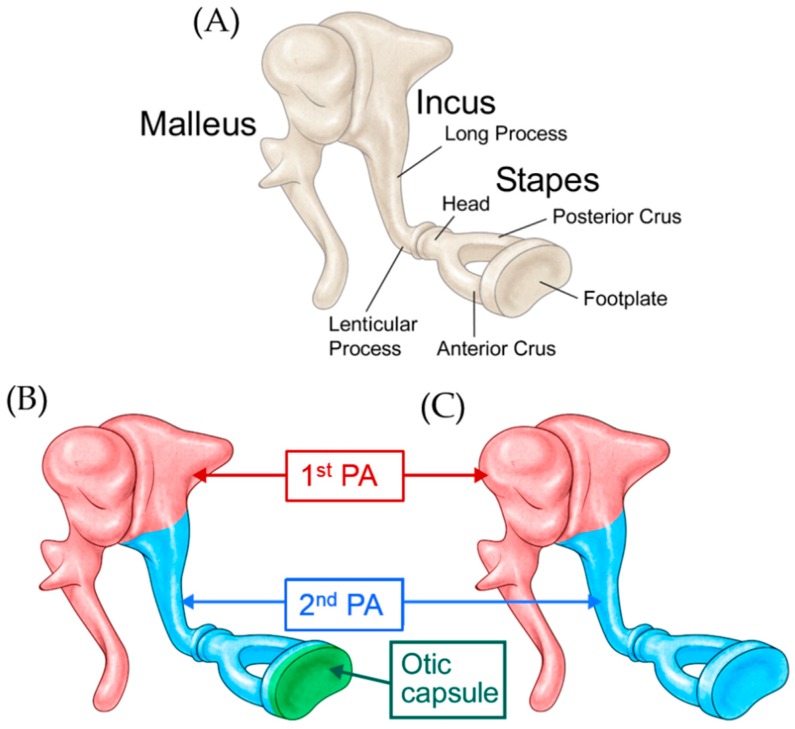
(**A**) Schematic representation of the ossicular chain; (**B**) Diagrammatic illustration of the theory suggesting that the medial portion of the footplate is derived from the otic capsule, while the lateral portion is derived from the second pharyngeal arch; (**C**) Diagrammatic illustration of the theory suggesting that the footplate is derived solely from the second pharyngeal arch. PA: pharyngeal arch.

A congenital ossicular anomaly should be suspected when a patient presents with nonprogressive conductive hearing loss with a normal tympanic membrane and no history of infection or trauma. Embryological studies of the development of the middle ear ossicles have shown that the head of the malleus and the body and short process of the incus are derived from the first pharyngeal arch, while the handle of the malleus, the long process of the incus, and the stapes suprastructure (the head and crus) are derived from the second pharyngeal arch [1] (Figure 1A). Early studies showed that the labyrinthine portion (medial half) of the stapes footplate is derived from the mesenchyme of the otic capsule, while the lateral portion is derived from the second pharyngeal arch [1,2,3,4] (Figure 1B). However, recent contradictory evidence suggests that the footplate develops independently of the otic capsule, being derived solely from the second pharyngeal arch [5,6] (Figure 1C).

Several studies concerned with classifying and predicting surgical outcomes in patients with congenital ossicular anomalies have been conducted [7,8,9,10,11]. Although ossiculoplasty is associated with favorable hearing outcomes in patients with congenital ossicular discontinuity and mobile stapes, unexpected intraoperative complications requiring further treatment may occur. In our institution, some patients undergoing ossiculoplasty for a congenital ossicular anomaly experienced intraoperative micro- or gross fracture of the footplate. The common findings in these cases were an absent or hypoplastic incus and a missing stapes suprastructure, indicating that the anomaly was associated with a defect in the second pharyngeal arch.

These unexpected intraoperative events prompted us to perform a retrospective study of patients who had undergone ossiculoplasty for ossicular anomalies associated with the malformation of the second pharyngeal arch. We hypothesized that ossicular anomalies related to the second pharyngeal arch would involve the lateral portion of the footplate, thus necessitating careful treatment. We assessed intraoperative findings and postoperative outcomes in these patients and summarized the potential complications and necessary intraoperative precautions. 

## 2. Materials and Methods

### 2.1. Study Design

This study is a retrospective analysis of patients who underwent exploratory tympanotomy and ossiculoplasty for a second-pharyngeal-arch-related ossicular anomaly between April 2015 and December 2018. The study was approved by the Institutional Review Board of the Clinical Research Institute of Seoul National University Bundang Hospital (IRB No. B-XXX), and the requirement for informed consent was waived. A review of the electronic medical records (EMRs), including surgical records, of 108 patients who underwent ossiculoplasty and exploratory tympanotomy during the study period revealed that 24 patients had undergone ossiculoplasty to correct an anomaly attributable to malformation of the second pharyngeal arch (i.e., anomalies of the long process of the incus and/or the stapes suprastructure). All of these patients were included in our study. Three experienced surgeons (JWK, BYC, and JJS (Ja-Won Koo, Byung Yoon Choi, and Jae-Jin Song)), each with at least 11 years’ experience as an otology specialist, performed all of the ossiculoplasty procedures. 

### 2.2. Clinical Data Collection

Clinical data, including demographic characteristics (such as age and sex), the side operated on, and the surgeon who performed the surgery, were obtained from the EMRs. The surgical records were reviewed to identify the types of ossicular anomalies, types of ossiculoplasty performed, and prostheses used. The pure-tone average (PTA) was calculated at 0.5, 1, 2, and 3 kHz for air conduction (AC) and bone conduction (BC) [12]. The air–bone gap (ABG) was calculated by subtracting the BC-PTA from the AC-PTA. The patients were followed up 1, 3, 6, and 12 months after surgery, and audiometric tests were performed at each follow-up visit.

All subjects underwent temporal bone computed tomography (TBCT) using a multidetector CT scanner (Brilliance iCT; Philips Healthcare, Best, the Netherlands) according to the protocol of our institution [13,14,15,16,17,18]. The imaging protocol involved helical acquisition with 120 kVp, 250 mAs, and a pitch of 0.825. Images were obtained at a slice thickness of 0.67 mm with 0.33 mm increments and reformatted at a section thickness of 0.7 mm with no gap. Both axial and coronal reformations were included in the image review. 

### 2.3. Statistical Analyses

Statistical tests were performed using SPSS software (version 17.0; SPSS Inc., Chicago, IL, U.S.A.). Paired *t*-tests were used to compare pre- and postoperative hearing thresholds; *p* values < 0.05 were deemed to indicate statistical significance in all analyses. 

## 3. Results

### 3.1. Demographic Characteristics and TBCT Findings

The study included 24 patients with ossicular anomalies due to defective development of the second pharyngeal arch. Of the patients, 13 (54%) were male, and 11 (46%) were female. The average age at surgery was 31.5 ± 15.1 years. In total, 11 (46%) surgeries were performed on the right ear and 13 (54%) on the left ear. Of the 24 surgeries, 13 (54%) were performed by JJS, 6 (25%) by JWK, and 5 (21%) by BYC. The mean follow-up duration was 10.5 ± 8.9 months.

On axial and coronal scans of TBCT, all subjects were found to have suspicious congenital ossicular anomalies involving the long process of the incus and the suprastructure of the stapes. However, due to the limited resolution of TBCT, we could not evaluate the risk of footplate fracture preoperatively.

### 3.2. Postoperative Hearing Outcomes 

**Figure 2:**
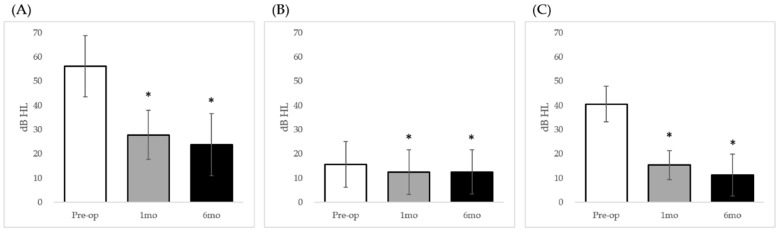
Changes in audiometric parameters after surgery. (**A**) Air conduction (AC) pure-tone average (PTA); (**B**) Bone conduction (BC) PTA; (**C**) Air–bone (AB) gap. * *p* values less than 0.05; Pre-op: preoperative average; 1mo: 1-month postoperative average; 6mo: 6-month postoperative average.

The preoperative AC-PTA was 56.0 ± 12.4 decibels hearing level (dB HL), and the BC-PTA and ABG were 15.7 ± 9.6 and 40.4 ± 7.4 dB HL, respectively. At the 1-month postoperative follow-up, the mean AC-PTA (27.6 ± 10.1 dB HL) was significantly lower than the preoperative value (*p* < 0.001; Figure 2A), the BC-PTA was 12.4 ± 9.4 dB HL, and the ABG (15.1 ± 5.9 dB HL) was significantly less than the preoperative value (*p* < 0.001; Figure 2B,C). Furthermore, significant improvements were observed in the AC-PTA, BC-PTA, and ABG 6 months after surgery (23.8 ± 13.2, 12.5 ± 9.4, and 11.3 ± 8.9 dB HL, respectively; *p* < 0.05; Figure 2). In total, 21 (87.5%) patients had achieved successful ABG closure (<20 dB HL) [19] at 6 months after surgery. Subjects with congenital ossicular anomalies who have undergone ossiculoplasty are routinely followed up until 12 months postoperation at our institution, but only 11 of 24 patients (45.8%) in the current case series were followed up until 12 months postoperation, probably due to subjective satisfaction and no further need to revisit the outpatients’ clinic after 6 months postoperation. The mean 12-month postoperative AC-PTA (26.6 ± 15.1 dB HL) and ABG (13.2 ± 9.8 dB HL) of these 11 subjects showed significant improvement as compared with the preoperative AC-PTA (57.6 ± 14.1 dB HL) and ABG (40.7 ± 8.2 dB HL) (*p* < 0.01). Meanwhile, the 12-month postoperative BC-PTA (15.7 ± 9.6 dB HL) of these 11 subjects showed a tendency of improvement as compared with the preoperative BC-PTA (16.8 ± 11.6 dB HL), but this difference failed to reach statistical significance (*p* = 0.089). The most commonly performed ossiculoplasty was total ossicular replacement prosthesis (TORP; *n* = 19), followed by partial ossicular replacement prosthesis (PORP), incus–footplate assembly (IFA), and malleostapedotomy. Although the stapes suprastructure was rudimentary in the two patients who underwent PORP reconstruction, it was sufficient to enable placement of the prosthesis. 

### 3.3. Intraoperative Complications

**Figure 3:**
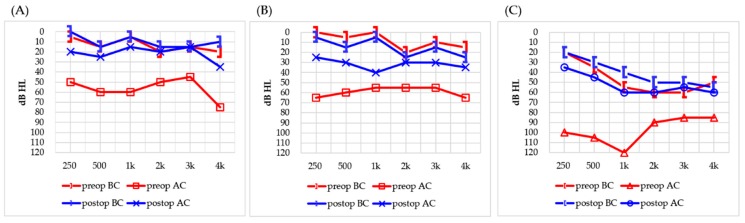
Comparison of preoperative (preop) and postoperative (postop) audiometric outcomes in patients with intraoperative complications. (**A**) Case 1, a 42-year-old male; (**B**) Case 2, a 35-year-old male; (**C**) Case 3, a 61-year-old female. BC: bone conduction; AC: air conduction.

Intraoperative complications occurred in three cases, although no patients experienced postoperative complications. All three cases of intraoperative complications involved fracture of the stapes footplate. The first case was a 42-year-old male who was missing the long process of the incus and stapes suprastructure. The footplate sustained a microfracture while its mobility was being checked, despite being manipulated in the conventional manner using gentle pressure. The fractured portion of the footplate was covered with tragal perichondrium and soft tissue, and a TORP was then placed on the reconstructed footplate. No change was observed in the postoperative BC thresholds, and the ABG improved from 40 to 12.5 dB HL (Figure 3A). The second case was a 35-year-old male with conductive hearing loss and a preoperative ABG of 47.5 dB HL. A malformed incus and a floating stapes suprastructure were identified intraoperatively. After the floating stapes suprastructure was removed, gentle pressure applied to the footplate to check mobility caused a fracture in the posterior portion. Because the defect was large and the long process of the incus was missing, a malleostapedotomy using a piston-wire prosthesis (length 5.8 mm; diameter 0.4 mm) was performed. No postoperative complications were observed in this case. The BC threshold remained the same, while the ABG improved from 47.5 dB HL preoperatively to 17.5 dB HL postoperatively (Figure 3B). The third case was a 61-year-old female with hypoplastic incus and stapes footplate and with no suprastructure identified intraoperatively. The footplate was fractured while checking its mobility. The damaged footplate was sealed using soft tissue and fibrin glue. After reconstruction of the footplate, a TORP (length 4.75 mm) was implanted. The ABG was reduced from 47.5 to 12.5 dB HL, with no change in the BC threshold (Figure 3C).

## 4. Discussion

We reviewed the intraoperative findings and postoperative outcomes of ossiculoplasty in patients with ossicular anomalies attributable to abnormal development of the second pharyngeal arch. Furthermore, we described three cases of intraoperative iatrogenic footplate fracture and the postoperative outcomes.

### 4.1. Postoperative Audiometric Outcomes in Subjects with Second Pharyngeal Arch Related Ossicular Anomalies 

Congenital ossicular anomaly is a rare cause of conductive hearing loss, with a reported incidence of less than 1 per 15,000 births [20]. In 1993, Teunissen and Cremers classified congenital ossicular anomalies into four categories, based on surgical findings in 144 ears with middle ear malformations: congenital isolated stapes ankylosis (Class 1), congenital stapes ankylosis in combination with a congenital anomaly of the ossicular chain (Class 2), a congenital anomaly of the ossicular chain accompanied by at least a mobile stapes footplate (Class 3), and aplasia or severe dysplasia of the oval window or round window (Class 4) [1]. This classification system was further refined by Park et al. [10] according to the presence or absence of the stapes suprastructure. A previous study found that the most common ossicular anomaly was a mobile stapes with a defective long process of the incus and a missing suprastructure, corresponding to Class 3 of the Teunissen and Cremers classification system [21]. Thus, it follows that ossicular anomalies resulting from malformation of the second pharyngeal arch are the most common anomalies because the long process of the incus and stapes are derived from the second pharyngeal arch. 

Several studies reported achieving significant hearing gains by exploratory tympanotomy and ossiculoplasty in subjects with congenital ossicular anomalies [11,19,22,23]. The rate of successful ABG closure (<20 dB HL) achieved at 6 months postoperation in our study following exploratory tympanotomy and ossiculoplasty, i.e., 87.5%, is comparable to that of previous studies, which reported closure rates ranging from 56% to 70% [11,19,22,23]. Although four types of surgery were performed in our cohort (TORP, PORP, IFA, and malleostapedotomy), the small sample size in each group (except for the TORP group) prevented us from determining whether one type was superior to the others.

### 4.2. Risk Factors Associated with Ossiculoplasty in Subjects with Second Pharyngeal Arch Related Ossicular Anomalies 

Of the 24 cases in our study, 3 (12.5%) sustained iatrogenic footplate fractures while the mobility of the footplate was being checked, requiring reconstruction. Although these cases were managed successfully, and the postoperative audiological outcomes were satisfactory, we investigated the possible reasons for frequent fractures of the footplate and the intraoperative precautions that should be taken to reduce the risk thereof.

**Figure 4:**
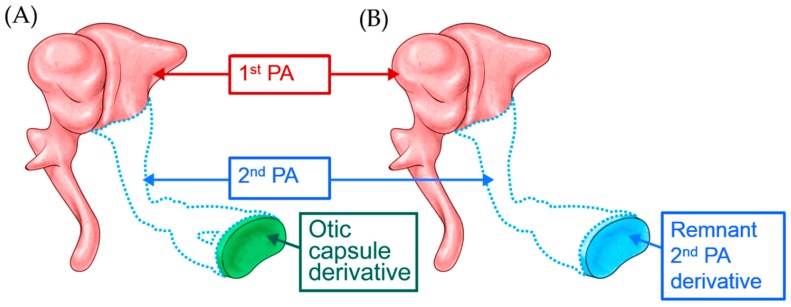
The stapes footplate is (**A**) partially or (**B**) totally derived from the second PA.

The developmental origin of the stapes footplate is controversial. While previous investigators have argued that the labyrinthine portion of the footplate is derived from the otic capsule, while the lateral portion is derived from the second pharyngeal arch [1,2,3,4], recent findings suggest that the entire footplate is derived from the second pharyngeal arch [5,6]. Nevertheless, given that the stapes footplate is at least partially derived from the second pharyngeal arch, it follows that patients with ossicular anomalies related to a defect in the second pharyngeal arch who are missing the long process of the incus and stapes suprastructure are likely to have an abnormally thin footplate (Figure 4A,B). Thus, when correcting ossicular anomalies caused by malformation of the second pharyngeal arch, surgeons should expect the footplate to be fragile and refrain from exerting excessive force when checking its mobility or implanting an ossicular prosthesis. Furthermore, surgeons should be prepared to perform malleostapedotomy or stapes reconstruction and TORP with ossiculoplasty in these patients.

### 4.3. Study Limitations and Future Directions

To our knowledge, this study is the first to report unique intraoperative complications of ossiculoplasty in patients with ossicular anomalies due to malformation of the second pharyngeal arch. However, our study had several limitations. First, although we have proposed several possible reasons for frequent footplate fracture, none have been histologically or radiologically confirmed, given the limited resolution of TBCT. Further assessment of footplate thickness using postmortem histological analyses or the high-spatial-resolution TBCT introduced in a recent cadaveric study [24] is needed to support our findings. Second, we can only speculate on the possible causes of a thin footplate, given that no consensus has been reached regarding the developmental origin of the stapes. It may be that future embryological studies confirming the origin of the ossicles will support our findings. Moreover, histological or embryological comparisons of cases with missing or mobile footplates versus those with no long process of the incus or stapes suprastructure may further our understanding of congenital ossicular anomalies.

## 5. Conclusions

Our findings suggest that patients with ossicular anomalies attributable to malformation of the second pharyngeal arch are likely to present with a relatively thin footplate. Thus, a fragile footplate should be expected, and care must be taken to avoid exerting excessive force on the footplate when checking mobility or placing an ossicular prosthesis on the footplate. However, when these complications are overcome, the audiological outcomes after exploratory tympanotomy and ossiculoplasty are excellent in most cases. Furthermore, with proper management, patients with an intraoperative footplate fracture can achieve successful ABG closure.
